# The Doctor and His Work

**Published:** 1912-10-19

**Authors:** 


### THE MAKING OF THE MODERN PRACTITIONER.

### The Doctor and His Work.

Several times within the last twenty years—and probably also in the preceding generation as well—books have been published with the avowed object of enabling the general public to understand the broad lines upon which medical practice is based; to explain that which the laity are so slow in grasping and so unwilling to accept—namely, that the science of diagnosing and curing disease is in truth just as rational as that of the engineer who applies himself to a broken-down motor-car. The popular idea that this science is a mysterious gift residing in the physician, who can exercise it without any necessity for asking questions or examining his patient if only he be "clever" enough, dies very hard. But by pegging away, medical authors may get lay readers to take it in some day.

We have spoken of the science of curing disease: there is an art of medicine and surgery as well. This art is decidedly more in the nature of a gift than is the science; it includes the quiet voice, the cheerful smile, the gentle hand, the aspect of firm decision and helpful sympathy. Let no one undervalue these things; but, on the other hand, let them not be confused with the true science of medicine. The art of medicine stands much where it did two generations ago; the science has made such enormous strides that it has to be rewritten constantly. To attempt to render intelligible to the public the methods by which this science is applied to their needs and necessities when they are ill is a task of difficulty, simply because of this ever-changing, ever-advancing progress in medical science. But it is a task worth doing, simply because an intelligent patient can help his own cure so very much if he understands in outline the main principles of his doctor's strategy.

A few years ago Mr. Brudenell Carter, the distinguished ophthalmic surgeon, published such a book. But matters medical have undergone such unforeseen developments since then that the time is ripe for a similar book dealing with the situation at the moment, complicated as it is by the Insurance Act and by the recent prominence of preventive as opposed to curative medicine. Such a book has just been published,* and though the title is almost the same as that of Mr. Carter's book, there is less objection to this than there otherwise might be because Dr. Whitby is a worthy successor.

Nor is it only the lay public who will profit by a perusal of Dr. Whitby's pages. The profession, too, may well lay to heart many of his wise and weighty sayings. For Dr. Whitby has a philosophic bent and a singularly broad and humane mind, as well as the pen of a ready writer. Not often, in truth, does the King's English fare so well in a medical book as in this case; for the author knows how to compose in a pure and scholarly style, which is also simple, lucid, and graceful—as indeed all English prose should properly be. His chapters make pleasant and easy reading, notwithstanding the concentration of thought which calls forth a constant exercise of the reader's intellect in response. In a word, the book is equally to be recommended to the philosopher, the doctor, and the educated public.

Dr. Whitby sees very clearly, and has the knack of making his readers see, the trend of events and the lines along which the evolution of the modern practitioner is proceeding. The rise of the specialist and the advantage of retaining the best sort of general practitioner as a check upon the narrowed horizon which only too often limits the former's field of vision, these are topics which others have discussed; yet even upon them the author finds things to say that are worth saying. His defence of the code of conduct known to doctors as professional ethics and to the public as "medical etiquette" is another of the themes which he handles dexterously; though here, we think, he presents only part of the case for the retention of this curious anachronism, as business men regard it.

Dr. Whitby's views upon the often-debated subject of unnecessary operations are thoroughly sane. Mr. G. B. Shaw and his *Doctor's Dilemma* are strongly and justly criticised; but Dr. Whitby does admit that the temptation to operate more for the sake of the fee to be earned than for that of the patient's benefit may occasionally (though very rarely) prove too much for a surgeon. He would meet this by a rule that in any case of a serious operation—we do not see why the "serious" should be insisted upon—the decision for or against its performance should not be accepted or even sought from the man who is to perform it. In actual working we do not think such a restriction would be workable; but the matter is one for argument and discussion.

The sanatorium, and its second cousin of an older generation, "the hydro," come in for sensible explanation in a chapter dealing with the "signs of change." A layman who reads this chapter will have a much clearer perception of what those institutions can do and what they cannot. On the topic of the moment, club practice, the author is entitled to speak with authority, and we wish the Chancellor and Mr. Masterman could read what he writes. For Dr. Whitby is by no means an unfriendly critic; he knows where the shoe of contract practice pinches, and that its fit is not likely to be improved by the Insurance Act in its present form. Eugenics, school inspection, and other similar medical and semi-medical schemes for bettering the nation are frankly and freely discussed, always with a strong common sense. It is this latter quality, with the delightful style in which the book is written, which is Dr. Whitby's transcending merit; and we trust that not only will the public absorb this book greedily, but also that the profession will lay to heart much of the high thinking and plain speaking which it contains.

* *The Doctor and His Work.* By C. J. Whitby, M.D. Stephen Swift. 1912. Pp. 235. Price 3s. 6d. net.

